# Notes on Medical Matters and Medical Men in Paris

**Published:** 1847-10

**Authors:** David W. Yandell

**Affiliations:** Louisville, Ky.; Paris


### THE WESTERN JOURNAL OF MEDICINE AND SURGERY.

OCTOBER, 1847.

**Art. I.**—*Notes on Medical Matters and Medical Men in Paris.* By David W. Yandell, M.D., of Louisville, Ky.

At the time of writing my communication on Etherization applied to allay pain in surgical operations, I promised another upon its application to midwifery. At that period but few experiments had been made, and no entirely satisfactory results had been obtained; for notwithstanding the marvelous achievements of ether had astonished the world, and inspired on all sides the most pleasing anticipations of advantages still to be realized from it, there seemed a moment when the minds of the most adventurous and eminent practitioners—of those, even, who had witnessed the triumphs of this agent in allaying, what may be termed *pathological pain*, were obliged to pause after the idea had been conceived of applying the powers of ether, no more to morbid cases, but to one of nature's most noble processes, that of parturition, from which pain, but in the *physiological* sense, is known to be inseparable. Analogy was questioned: Was pain not always pain whenever it caused the human frame to shudder or cry for relief? Curiosity urged—was the domain of this marvelous vapor to be limited to the surgical art so far only as the ablation of limbs and the division of living tissues were concerned? Reason demanded—would the antidote so signally successful against pain when produced by the bistoury, fall powerless when opposed to the pain concomitant upon the performance of certain duties of nature? The solemn laws of the healing art, and the philosophical precepts of physiology, both tended to make those who had been the most enthusiastic in regard to the conquest already gained, pause, and consider the justifiableness of the one they were about to attempt. Things could not long remain thus. Fortunate and daring leaders were upon the bank; the dangerous stream glided peacefully by; they plunged in, and obstetricians have perhaps advanced farther towards the source of nature's mysterious action than their surgical brethren have been called to do, in as much as, in the question of etherial inhalation, the former have not had to interfere with the diseased economy, like the latter, but with a physiological phenomenon at the highest degree of action. And to the honor of obstetricians be it said, they have acted with the most praiseworthy reserve, and with a due reverence for nature whenever they have employed this agent during parturition. Indeed it is to the prudent investigations of the delicate question in hand,—the intervention of an agent abolishing pain in labor, that we are indebted for, we may say, the first regular system of well planned and well conducted experiments on etherization. Enthusiasm and confidence reigned supreme at first. When nature became more deeply involved by the attempts made to modify her ways, cool reason, prompted sometimes perhaps by a growing timidity, assisted to temper this interest, to awaken a calmer spirit of inquiry, and compel a more rigid and safe method of research. Theory and practice have both obtained a certain degree of advancement and certitude in the conflict. The phenomena displayed during the parturition of etherized women have elicited interesting questions relative to the action of the cerebro-spinal ganglionic system; it now remains with the physiologists to pursue their researches in that direction. Pain has been abolished for a certain period of labor, without any apparent disturbance having arisen throughout nature's course. These practical facts have been principally illustrated by the elaborate and truly scientific communication to the Academy of Medicine of Paris, by Prof. Paul Dubois, and by the notes published by Prof. Simpson, of Edinburgh. New facts have since then been contributed by other accoucheurs in France, whose observations we shall relate in the order in which they have appeared, thereby, possibly, accomplishing in as succinct a way as any other the object of this paper, which is to give the American reader an idea of the actual state of etherization applied to midwifery in this country. I shall begin, after a few preliminary remarks, by a detailed account of Baron Dubois' communication to the Academy, since it continues to be the principal scientific document on the subject before us.

For this account, as well as for most of the facts contained in this paper, I am indebted to my friend, Dr. Charles J. E. Campbell, who is already favorably known to the profession both in England and Paris as a promising young accoucheur. I cannot adequately express my thanks to him for the kindness which he has displayed in placing his note-book at my disposal. These notes were taken at the sitting of the Academy of Medicine, and were further extended at Prof. Dubois' lecture upon the inhalation of ether.

The two noted champions of obstetric science in Paris and Edinburgh, Prof. Dubois and Prof. Simpson, proceeded about the same time, but on very different plans, to observe the phenomena offered by ether administered during parturition. Prof. Simpson presented to the profession certain facts relative to etherization, as they occurred to him in his practice, while Prof. Dubois established at the Maternité, and at the Hôpital des Cliniques, a series of experiments, with the preconceived intention of ascertaining the exact value of the method when employed in those special cases; the one, therefore, purposing, by some cursory observations, to throw out to the world the assurance of the applicability of the method; the other evidently determined to analyze the character, value, and bearing of the process when adapted to midwifery practice. In this procedure of the veteran French obstetrician, we have another evidence of the sagacious and philosophical spirit which forms the distinguishing trait of the Professor of obstetrics at the Maternité of Paris.

Baron Dubois, as expressed by himself in his communication to the Academy of Medicine, on the 23d of February last, felt greatly embarrassed when he began his investigations concerning etherial inhalation in parturient women. Up to the period we have just stated, there existed but few allusions to the matter, and but three instances were known in which ether had been employed in the practice of midwifery. Dr. Bouvier, in the Academy of Medicine, and M. Velpeau, at the Academy of Sciences, while speaking of the influence of this agent in medical and surgical cases, had merely expressed the opinion that it was not improbable, that the use of ether would be found efficient in certain cases of the obstetric art. The surgeon of La Charité had so far extended his views on this point as to say that, independently of the well known advantage of preventing pain, the agent might probably possess the power of diminishing certain muscular resistances in difficult labors, such as that of the contracted uterus in cases where turning the child became necessary. There is one case known, recorded by Prof. Stoltz, which proves, as far as one case can prove it, that this anticipation is not as yet realized.

As to the three facts to which I have adverted as existing at the time when Baron Dubois began his inquiries, the first, constituting a kind of transition from surgical operations to those necessitated by difficult labor, is one in which the cæsarian section was performed by Dr. Skey, of Bartholomew's Hospital, in London, on the 23d of January, and which was but imperfectly reported at the time. The second case, anterior in date to the first, is one of turning, performed by Prof. Simpson, on the 19th of January, and given in the notes published by the Professor on the 22d of March. The third was a case of application of the forceps, in the etherized state, performed by Dr. Des Champs, and published in the *Gazette des Hôpitaux.* With these few precedents to be found at that period in this department of science, and amidst the most sanguine expectations on the part of the scientific world as to the event, Prof. Dubois commenced the investigations now about to be related.

The Professor began by an allusion to the feelings which naturally and unavoidably arose in the mind of one who, unprecedented as it were, had to enter a field of phenomena never witnessed before; and, after dwelling upon the difference of his position from that of the French surgeons, who had instituted their operations after the American and English surgeons had communicated to the world the result of their experience, he proceeded to divide his inquiries into the following heads:

1. *Can ether be the means of preventing pain in operations of midwifery?*

2. *Can ether suspend and annihilate the physiological pains of natural labor?*

Before attempting to give a direct solution of these principal questions, Prof. D. deemed it necessary to investigate the following important points, the elucidation of which will furnish the best elements for a proper answer to the whole of the inquiry. Thus, he desired to ascertain *whether ether could be prejudicial to the mother primarily? If it could inflict any injury on the child also?*

*Whether this agent, after having exerted a stupifying action on the cerebro-spinal nerves, would not induce a paralyzed state of the uterus?*

*Whether being innoxious primarily, ether might not, consecutively, act on the constitution of the mother or of the child?*

As to the action of ether on the child, no one, said Prof. Dubois, can entertain a doubt, considering the diffusibility of its vapor, that when introduced into the mother's system by a large and active surface like that of her respiratory organs, it may pervade the child's system also, by means of those vascular communications existing between the mother and the fœtus. It was natural to examine the very delicate question of the possible amount of this influence, and it will be acknowledged, after hearing the facts I have to relate, that my anticipations have been realized so far as the penetration of ether through the system is concerned, but not in the least, I am happy to say, as to the dangers thereon attendant. On examining the question of the effects of ether on the mother, we find arguments of a special nature, drawn from facts already noticed, during the inhalation of ether in other circumstances. Cases have been witnessed in which the precursory signs of intoxication have consisted in an involuntary, disorderly, and convulsive agitation of that muscular system which is subservient to volition; in some cases also, the intoxication induced by ether has been itself characterized by a kind of epilepsy, or catalepsy, and has either been accompanied, preceded, or followed by convulsions. Now, if through life, we look for a physiological condition in which there may be found a predisposition to phenomena of this kind, we shall remark, undoubtedly, that pregnancy itself acts, but too frequently, as a predisposing cause to that species of nervous excitement which, in some cases, brings the subjects to a fatal end. I allude, as will be understood, to *puerperal convulsions.* It is therefore manifest that ether, during the pregnant state, cannot be exhibited with too great circumspection. In the course of this communication I shall have occasion to relate a case which came under my own notice, and which partially confirms the natural apprehension that ought to be entertained on this subject. This apprehension is moreover corroborated by facts observed in medical practice. Thus, Prof. Piorry brought before the Academy, at the last meeting, cases of hysteria in which he administered ether as a sedative, and in which the fits, far from being changed for the better, were, on the contrary, rendered more intense, epileptic phenomena having been induced. Another example, illustrative of a similar effect, is founded on the fact of a physician, acting on the same preconceived notion of the sedative powers of ether, having administered this agent to patients laboring under epilepsy, and the attacks having been thereby renewed with far greater intensity than before. And again, it appears that ether was tried in England in a case of tetanus, for the same purpose; namely, of subduing nervous irritability and muscular contraction, but without any successful result. It fortunately happens, however, that in our cases those apprehensions have not been confirmed by accidents of this nature.

Having thus laid before the Academy the scrupulous considerations under which he labored for some time, and which he still desires should be maintained in the minds of the profession, on account of the very small number of experiments hitherto madê on the subject, Prof. Dubois next proceeded to examine the principal question. 1. *Does ether prevent pain during obstetrical operations?* This he illustrated by cases, as follows:

Case I. A woman æt 18, primipara, entered the hospital of the Maternité, February 15th; labor had already lasted 28 hours; the uterine contractions were feeble, slow, and of an intermittent nature. I thought I should probably accelerate the pains by perforating the membranes. I did so, but no change taking place ergot of rye was given; but this again was not followed by the desired result. It was then that I decided on applying the forceps, having previously placed the patient in a state of insensibility by the inhalation of ether. This was accomplished with difficulty, the woman seeing no necessity for it, and being disturbed by the inhalation which she said was very disagreeable. In the course of six minutes, however, she fell into an apparently insensible condition. I then attempted to introduce the first blade, but at that moment she made a short, abrupt motion with one thigh. I therefore postponed the introduction of the instrument and caused the patient to resume inhalation for two minutes; after which a kind of soporose quiet was brought on, and the first blade was introduced; the placing of the second blade, the prehension of the child's head, the locking of the forceps and the extraction of the child proved very easy. *During all the manœuvre there absolutely was not the least pain.* The expanding of the perinæum and the dilatation of the vulva were so rapidly effected, that I was enabled to terminate labor in a shorter time than nature generally takes to complete the process after it has arrived at the same stage. The child was living. The cord beat 160 times a minute, but within a few minutes after birth the pulsations of the child's heart fell to their normal state—from 136 to 130 per minute. Here we may inquire whether the child had suffered from long labor, from the application of the forceps, or from the ether? I shall presently allude to another case in which the same derangement in the fœtal circulation took place, and where it could not be attributed to long labor, or to any interference of art, the case being a natural one. In the present case, the woman very soon regained her consciousness, and being asked, 'are you delivered?' she stretched both her hands down across her abdomen and answered—'Why, sir, I see I am.' 'Did you suffer at all?' 'No sir'—'although,' said she, 'I felt as if I were surrounded by attendants who came here to deliver me.' This woman had evidently been in a state of complete insensibility, though of imperfect consciousness. The dull perception she evinced of what had been going on around her may be attributed to her having witnessed, before inhaling the ether, the preparatory disposition made for her delivery.

Case II. A woman æt 19 years, having reached the term of pregnancy, came to the Maternité with lingering pains that had existed for thirty-six hours. She felt greatly fatigued. I considered this a proper case for using the forceps. The patient was made to inhale the ether for ten minutes, a few interruptions having necessarily taken place, after which apparent insensibility came on. I began applying the instrument, but the movements and loud cries of the woman at that moment induced me to readminister the ether. We did so for five minutes, when stertorous sopor was brought on, during which the forceps were easily applied. The patient then began to be somewhat agitated and uttered shrieks; delivery, however, was rapid. The perinæum appeared to stretch out with greater facility than usual. The woman then seemed to become conscious of what was going on, when I said to her—'Are you delivered?' She answered, 'yes.' 'Did you suffer?' 'No,' 'Do you remember having felt any pain or uttered any loud cries?' 'No, sir, I can remember nothing of the sort.'

The Academy will notice, said M. Dubois, that in these two cases ether produced different effects, sensibility having been annihilated without the suspension of consciousness, in the first case; whilst, in the second, sensibility was affected in the same degree as in the first, with the difference that consciousness was also totally abolished. Facts of this nature have already been so often presented to the attention of the Academy, that I deem it unnecessary to dwell any longer upon them. The only thing I wish to observe is, that both cases, but especially the first, tend to establish the proposition, that ether may be employed in the practice of midwifery, as it has hitherto been in surgery; that is, to prevent pain during obstetrical operations.

2. *Can the inhalatian of ether suspend the physiological pain attending natural labor; and can it suspend the contractions of the uterus and of the abdominal muscles?*

These questions, said Prof. Dubois, are not only of vast experimental and physiological import, but give rise to practical views the value of which no one will be inclined to doubt. The physiological point of the question is, to ascertain whether or not the stupifying action of ether on the cerebro-spinal axis, and the paralysis of the muscular system under the control of the latter, will extend to the uterine parietes. At this period, remarked the professor, the question of uterine innervation is still an unsolved problem. Scientifically speaking, it stands thus—the uterus receives its nerves, perhaps exclusively, as is the opinion of Longet, from the ganglionic system, on which, however, the spinal cord may exert certain influences arising from the communications existing between the two. Nevertheless it remains an evident fact, that the uterus is an organ the contraction of which can neither be suspended nor in any way modified by volition. This fact implies that this organ receives its nerves from the ganglionic system; and if we admit the latter to be influenced in its action by that of the cerebro-spinal axis, it would probably be found that the origin of that influence is not direct. If these anatomical considerations be true, ether would appear to act exclusively upon the voluntary organs of animal life, and therefore ought not to influence the state of contractions which take place when prompted by organic vitality. I am aware that a distinguished physiologist, M. Brachet, is of a contrary opinion. He thinks the uterus is subject to the direct influence of the spinal cord. In support of his views he cites certain experiments on animals, and a morbid case observed in the human species. The experiments consist in vivisections performed at various points of the spinal cord, after which, says M. Brachet, the uterus becomes inert. The pathological fact is one of paraplegia observed in a pregnant woman. I am not prepared to gainsay the physiological facts; but concerning the case of labor in the paralytic woman, it is far from possessing the value M. Brachet would wish to claim for it; since it is asserted that an application of the forceps became necessary to help labor, at a moment when the dilatation of the orifice would allow the operations to be performed without danger. Now, I will only say this—that if in the alleged case the orifice was dilated, the dilatation could have taken place by no other cause whatever except the influence of uterine contraction. It is probable, however, that M. Brachet's opinion has in some measure prevailed among the scientific world; for many learned medical men have inquired of me recently whether ether did suspend uterine contraction. I am far from pretending in any way to decide these difficult questions. The facts I

am about to lay before the Academy have appeared to me of such a nature as to throw much light upon them; in as much, at least, as the effects produced on the nervous system, the brain and the spinal cord, may be considered a trustworthy criterion. Let this, however, be decided as it may, these cases will not prove the less curious or interesting from being seen under various other points of view. I have already mentioned, and I now recall to mind, a remark I made in the first forceps case related; namely, that at the very moment I attempted to introduce the first blade, the woman had made a kind of sudden effort which succeeded in impeding the introduction of the instrument. I now proceed to far more conclusive facts.

Case III. On the 5th of February, at 10 o'clock, A. M., I saw, in the amphitheatre of the Maternité a woman pregnant with her first child, who had reached the term of utero-gestation. She had been in pain since two o'clock that morning. The membranes were not ruptured; the dilatation of the orifice measured something more than one inch. Uterine contractions caused pains of an intense nature, which rapidly succeeded each other. To this woman we offered an immediate and total removal of pain. She immediately consented to inhale ether. The process of inhalation was occasionally interrupted by the woman becoming unmanageable during the moment of contraction; it lasted in all 25 minutes, after which she relapsed into a state of complete insensibility. At this period we witnessed a most curious and instructive though highly perplexing, phenomenon—determination of blood to the head. Her face became intensely red; her looks were set, her eyes being fixed upwards and outwards; the conjunctiva was congested to such a degree that I really could imagine blood on the point of issuing from its surface. The under lip was hanging, the tongue tinged, and spumous saliva issued from the mouth. This state lasted for three minutes. Squeezed and pricked during this period, the wowan felt nothing. During the state of insensibility, I remarked that the contractions continued, the uterus becoming globular and hard under my hand laid on the abdomen, while, *per vaginam*, my finger felt the tense bag of membranes protruding through, or rather against the orifice; these contractions were not painful in the slightest degree. Rather preoccupied with the above described state of the woman, I let, no doubt, a few contractions escape my notice, but there were two contractions which I perfectly observed and felt. After three minutes the woman recovered her senses and told me that she had not experienced the slightest pain. During the period of insensibility I had laid my ear on the abdomen, and counted the fœtal heart beating 160 times per minute. Very soon after the woman had recovered her senses I found but 140 pulsations, and two or three minutes later they had decreased to 136, the normal state; so that, in this case, as I have already stated, when alluding to it I spoke of the effects of ether on the child, the modification of the fœtal circulation could not be attributed to any other cause than the fact that the mother had inhaled ether.

Case IV. A woman æt 19 years, with child the second time, and arrived at the end of pregnancy, had been in labor since morning; I saw her at 3 o'clock, P. M., when she experienced very intense pains and uttered excessively acute shrieks. Such were her pains that she could not be made to understand that it was in her power, if she chose, to restrain, in some measure, her piercing cries, it really seeming to us that her physical powers alone could fix a limit to her wild and frantic screams. To this woman also we offered ease, and insensibility to her violent pains. To this she lent a ready ear, took the ether, and in three minutes fell into a deep soporose state; the eyes were open and set. She then quietly turned her head towards the female attendants around her bed, and beckoned to one of the midwives to draw near and kiss her. During the period of insensibility the contractions continued violent and energetic; they came and passed away in the deepest silence on the part of the woman. The bag of liquor amnii protruded against the finger introduced per vaginam; the fundus uteri hardened under the hand placed on the abdomen, and, nevertheless, there existed a calm, death-like state of the patient, that offered, when contrasted with the energetic process going on, one of the most striking and unusual phenomena ever witnessed in the practice of midwifery. I then ruptured the membranes and instantaneously a muscular effort was made, by means of which the head plunged deep into the pelvic cavity. At this advanced period of labor the patient awoke, and parturition very soon ended amidst the ordinary series of painful phenomena. After the expulsion of the fœtus the mother thanked me mildly for the relief I had afforded her. When I asked her how she felt, and whether she remembered what had passed, she answered, she felt very well, and that she 'had dreamt.' What did you dream of? was my inquiry; but the patient turned her face aside with a smile, the peculiarity of which having attracted my particular attention, I resumed the question. On her again refusing to let me know the nature of her dream, I had recourse, in order to ascertain it, to the intermediate communication of a reputable person of her own sex, who had assisted in the administration of the ether. To the same question, proposed by this person, she answered, that she had dreamt she was beside her husband, and that he and she were engaged in those preliminaries which had led to the state in which we now beheld her.

It now remained for me to ascertain whether this kind of ebriety, the effect of which had hitherto been to prevent pain without interfering with uterine contraction, could impede, or not, the action of the abdominal muscles.

Case V. A woman æt. 18 years, came to the Hôpital des Cliniques a few days ago. Labor was in an advanced stage; the bag of waters had given way; dilatation was complete; the head deep in the pelvis. The uterine contractions in this female were very painful indeed. She readily consented to be etherized. After ten minutes she became soporose, complaining of ringing in the ears, and saying she felt herself dying. Uterine contraction, together with the action of the abdominal muscles, continued throughout in a most evident manner; the uterine action becoming more and more frequent, the head and body of the child very easily and promptly cleared all the obstacles to their passage, and that without producing the slightest manifestation of pain in the woman. In this case I remarked the singularly relaxed state of the lower extremities, which had been placed, before etherization was resorted to, in the semi-flexed, angular position, according to the French custom. The moment insensibility was induced and the knees were no more supported by the hands of the assistants, the two limbs fell outwards, flat on the bed and in such a relaxed state, that they had a tendency to rotate still farther outwards had not the coxo-femoral joint proved an impediment to this exaggerated movement. The child was living, and it was its cries that awoke the poor mother, whose first thoughts and words were: 'Is it a boy or a girl?' The patient assured me she had not suffered in the least, only that it had appeared to her that she had been in the act of evacuating her fæces.

I must here subjoin a fact, which I have commonly observed in the course of my researches on the action of ether in those special cases—it is the extreme apparent laxity induced in the muscles of the perinæum. Even with primiparæ, the dilatation of those parts has been so rapid, that in the cases where parturition took place during the state of insensibility, the duration of which never exceeded fifteen minutes, the expulsion of the child has been painless; and we must remark, as worthy of notice, that one of these women bore a child weighing eight pounds, and that in her case the perinæum was not damaged in the slightest degree. I must also make the very important remark, that the uterus, after delivery in all the cases, has constantly and immediately resumed the firm, contracted state of its retracted parietes.

A last and very important question shall conclude my researches on this subject. I allude to the *consecutive effects of etherization on those patients.* None of the women who inhaled ether have experienced bad effects attributable to this agent. One of them felt her fingers benumbed for twenty-four hours; another complained of her legs being slightly benumbed also, and for about the same length of time; after which this feeling entirely disappeared in both cases. We noticed no cephalalgia whatever, no bronchitis, no nervous symptoms of any kind. Nevertheless, two of these women died of metro-peritonitis, and precisely those two who underwent the application of the forceps. I have put to myself the question, whether the exhibition of ether did in any way contribute to the fatal result in those cases? On questions so imperfectly elucidated as those relating to the influence of ether on the animal system, and to the etiology of puerperal disease, all kinds of hypotheses may be advanced; still it appears to me that, having conceded to the doubts that may arise all the weight they deserve, we should inquire whether any other circumstances were present, apart from the inhalation of ether, that might explain, in a more natural and satisfactory manner, the serious results to which I have alluded. Should we not inquire, for instance, whether the difficulties of parturition did or did not exert any deleterious influence in the cases? Then, are we not bound to ascertain what may have been at that moment, the sanitary state of the hospital? Whether the patients, who had not been etherized, were at that period equally subject to puerperal fever? Whether mortality prevailed, and to what extent, in all these cases? And last of all, whether the *post mortem* appearances in these two females were similar to those generally observed in the bodies of such as have died of the disease?

In answer to these questions I will say, that the delivery of the two women who died did take place under circumstances that may easily account for death. The application of the forceps in itself was prompt and easy, but one of the patients had been in labor 40, and the other 38 hours; such, we are well aware, are very unfavorable preliminaries to delivery, and in these cases they proved the more untoward from the circumstance of the Maternité being at that period under the influence of a slight epidemic puerperal fever. I may add, that the disease was equally prevalent among those patients who had not inhaled ether; that mortality prevailed to a great extent; and lastly, that the morbid lesions found on the *post mortem* examination of these two cases, were precisely and exclusively the same as those observed and recorded on the examination of other unetherized patients who died in the hospital about that period. We need not hesitate, therefore, between the choice of gratuitous hypothesis and well authenticated facts. We are bound to decide that ether, up to this moment, is not chargeable with any fatal results, and from the foregoing observations on the subject of this agent, considered in its applications to cases of midwifery, I feel myself justified in drawing the following conclusions: First, that the inhalation of ether has the power of preventing pain, during obstetric operations. Second, that it may also momentarily suspend the natural pains of labor. Third, that the state of ebriety induced by the inhalation of this vapor does not suspend uterine contractions when the latter have decidedly set in and take place at short intervals; and that it does not impede the synergetic action of the abdominal muscles. Fourth, that the state of ebriety appears to lessen the natural resistance which the perinæal muscles oppose to the expulsion of the head. Fifth, and finally, that the inhalation of ether has not appeared to exert any bad influence over the life or health of the child.

Now, after hearing the foregoing conclusions it may appear natural to suppose, that the inhalation of ether being a process to which so many advantages are attached, it is a precious expedient, to be frequently resorted to in ordinary cases, by the obstetrical practitioner. Such, however, is not my opinion. The very proposal of such a thing, having no other ground than the very few facts I have communicated to the Academy, would not only appear extremely bold, but should be considered as eminently imprudent. In beginning this communication I expressed the apprehension under which I labored at first; well, the cases I have brought before the Academy may have lessened my fears, but they have not yet altogether dissipated them from my mind. You will remember that one of the women who inhaled ether went into a state bordering upon that which we designate by the term *epileptiform.* Two other women died. Now, although the morbid occurrences in the first case were of but short duration; although the fatal result, in the two other cases, arose far more probably from the influence of the epidemic than from any other cause; still, the Academy will feel that in a question of so serious a nature, the recollection of these facts ought to leave on my mind impressions of doubt and timidity. Later, perhaps, these hesitations may disappear; but even then I shall not forbear thinking, that the very nature of things must render the exhibition of ether, in ordinary cases of midwifery, a matter of very doubtful propriety. First of all it is evident, that the inhalation can never produce painless labor, from beginning to end. It is to be doubted whether insensibility could be made to last long enough for such a result; and it is more doubtful still whether such an attempt could be made without incurring positive danger, and without being amenable to the charge of criminal temerity. There remains therefore for employing ether, only the last period of labor, as in the cases where I have used it; and even then, this period, during which its exhibition has appeared the most effectual, is the one, according to all mothers the least fatiguing, the least painful of all the stages of the process of parturition.

As to the use of ether in cases where it is necessary to resort to instruments, I will only observe, that operations of this kind are often rendered indispensable by unforeseen circumstances, and that when this necessity occurs, the cases are of a very urgent nature. It is not necessary to give further developement to the propositions I lay down here, in order to show that in a great number of such cases inhalation of ether cannot be resorted to. As to the remaining cases, it may be asked whether they prove to be generally of so painful a nature as to justify the common use of a process which, even in a condition unconnected with the puerperal state, is not free from disadvantages, and which, when used under those special conditions, seems to me still less free from danger. My profound conviction on the subject is, that inhalation of ether in midwifery should be restricted to a very limited number of cases, the nature of which ulterior experience will better enable us to determine.

Such was the first scientific communication made on the subject of the inhalation of ether in the practice of midwifery. Prof. Dubois has the intention, at some future time, to publish a memoir on the subject. In the mean time some additional cases have been recorded by M. M. Chailly, Bourier, Stoltz, and Danyan, which we now proceed to notice.

This memoir, by Prof. Dubois, was, as we have already stated, the first systematic attempt made in France towards ascertaining the action of etherial inhalation when applied to the practice of midwifery; and we believe the scientific world will esteem it the most important, since it gives a solution of most of the problems connected with the use of this agent. The remaining facts that have been published in Paris may so far contribute to advance the history of etherization as they serve to confirm what Dubois has already advanced, or show certain peculiarities of its action in special cases. Those facts may be given in a few pages. At the sitting of the Academy of Medicine on the 9th of March, M. Bourier related the following case:—A woman æt. 26 years, the mother of four children, entered the Hôpital Beaujon, at the full term of her fifth pregnancy four hours in labor. The pains were strong, and intense contractions took place every three or four minutes. The orifice was dilated to the size of a five franc piece. The woman was made to inhale ether about an hour before the probable period of delivery; she became agitated and fell asleep after eight minutes. M. Bourier was of opinion that uterine contractions then ceased completely—the suspension of pains lasted ten minutes, after which slight contractions returned, and three quarters of an hour after this, the pains had resumed their decided character of strength, and become more and more rapid in their recurrence. Delivery took place two hours afterwards. M. Bourier was of the belief that, in the foregoing case, ether was the means of impeding the progress of labor by interfering with the uterine action; and hence concludes, that ether may suspend those contractions when they have not arrived at the last period.

Now, one may inquire, whether the case observed by Dr. B. is not one of spontaneous cessation of pains, such as is often witnessed in the beginning of labor; or whether the suspension was not the direct and immediate result of the inhalation of ether? We feel it to be difficult to give a satisfactory answer; but in view of the two first cases related by Prof. Dubois, in which the pains ceased after long labor, and before delivery was accomplished, we must own that we are inclined, as MM. Roux and Danyan have been, to adopt the first explanation.

At the same sitting of the academy, Dr. Chailly reported the case of a lady, in private practice, who presented the most unusual sensibility of the vaginal orifice. The vulva was found to be somewhat constricted which had been the case since the last labor, seventeen years before, the lady now being 43 years old. There existed also a slightly contracted pelvis. As application of the forceps became necessary after a labor of 59 hours, ether was given, the operation was effected *without pain, without the slightest laceration of the perineum, and without any ulterior accident to mother or child.* The details of this interesting case are to be found in the Bulletin of the Academy of Medicine, and also in the *Union Médicale* of March 1847.

In the *Gazette Médicale de Strasbourg*, of March 27, 1847, Dr. Stoltz relates a case which somewhat elucidates that part of the question left unsolved by the experiments of Prof. Dubois, and alluded to by M. Velpeau at the Institute; viz: whether etherial inhalation could suspend or diminish uterine contraction in certain cases, those the most difficult in midwifery—where it is necessary to turn the child in a contracted uterus. No case of the kind had occurred to Prof. Dubois, but as he had proved that ether exercised no influence over normal contractions, it was natural to believe that it would not advantageously modify the state of the uterus during pathological contractions. However experience had not warranted the conclusion, Prof. Simpson's case of turning being one of contracted pelvis, when Prof. Stoltz met with a case most favorable for putting the method to the test.

A woman, æt. 24 years, came to the Clinique d'Accouchmens of Strasbourg on the evening of the 4th of March. The membranes had been ruptured since the preceding day; the uterus was strongly contracted, irregular in its figure, and tightened around a child, which presented the right arm, the right foot, and a loop of the umbilical cord without pulsation. Professor Stoltz after having brought the patient into a state of insensibility by the exhibition of ether, seized the presenting parts, but tried in vain to extract the others. The uterus, during etherization, offered an insurmountable resistance. He therefore undertook to extract one foot alone; it was only after some time that he was able to place his finger, in the manner of a hook, on the left groin, and thus he proceeded to extract the child as far as the head. It was then that he experienced the greatest amount of resistance. The woman was left to repose an hour. At last a few contractions took place, and new efforts being made, which nature seconded, delivery was easily performed. This is a very conclusive case, and the question now appears to be definitively resolved: *Etherization does not suspend either normal or pathological contractions of the uterus.*

In the May number of the *Revue Medico-Chirurgicale*, M. Danyan, professor of midwifery at the Maternité, has published a paper on the employment of ether in obstetrical practice. He presents the reader with an admirable summary of the experiments hitherto made on the subject, but adduces no new facts of his own personal observation. He introduces into his paper a passage from Longet's remarkable memoir on the subject, which is as follows: "In the midst of the general and profound collapse, says Longet, in which the organism is involved, and of the proximate danger which threatens it with destruction, an attentive sentinel is still awake. This vigilant agent of protection is the organ which constitutes the primary motor of the respiratory mechanism, —it is the rachidian bulb. On it depends the maintenance of respiratory motion, and also the dilatation of the nares and mouth, the opening of the glottis, the elevation of the costal and scapular regions, the contraction of the diaphragm and of the *abdominal muscles*, inasmuch as they are considered muscles of respiration. Now, *effort* in general, and particularly that which accompanies parturition, is but a modification, a momentary change of the respiratory function; it is a state during which there must exist energetic contraction of the costal and scapular muscles, of the diaphragm, of the muscles of the abdominal parietes; during which, as Isidore Bourdan and J. Cloquet have justly observed, the glottis is spasmodically constricted; during which many other muscles also contract by that law of synergetic action on which Barthés has written his numerous and valuable papers. If, during etherization, in the absence of volition, respiration still goes on in all the integrity of the function, and if the bulb continues exciting the muscles which accomplish the act, the effort which is the result of the contraction of these identical muscles (those of the abdomen included) must also, it follows as a consequence, take place; for if, in the majority of cases, the muscular contractions which produce an effort are under the influence of volition, there are some instances in which they appear to be independent of the will. It is precisely this exception which we are called upon to witness at a certain period of labor, in certain operations, as lithotomy and lithotrity, in which the contractions of the uterus or bladder are irresistibly followed in their action by that of the abdominal muscles, diaphragm, etc. As to the perineum, if it ceases its contraction in parturient women under the influence of ether, as Professor Dubois has recently observed,—if its natural resistance is annihilated, and if it falls into the general collapse of the muscles of animal life, it is because the perineum does not constitute a part of the respiratory muscular apparatus, as we see that the abdominal muscles do. It is because during the effort, which is of an involuntary kind, it is merely depressed under the weight of the abdominal viscera, and only opposes to the effort a *vis inertia*, principally through the medium of its aponeurotic layers. I allow, on the contrary, that during an effort directed by the will, the perineal muscles will contract, but in the same manner as many other muscles that are not under the direct influence of the respiratory centre, and simply in accordance with the law of synergy to which I have already alluded."

Dr. Campbell has taken the deepest interest in the subject of etherization in obstetric practice, and I feel very sure has made a faithful report of M. Dubois' admirable memoir. I regret that I could not avail myself of the advantage of seeing his report in the London Lancet before completing my own translation. I am persuaded, however, that the inherent interest of the subject will render your readers comparatively indifferent to any faults of style, which may be detected in a hasty performance.

A curious question is frequently propounded to those who propose employing ether to abolish the pains of labor, which is, can such an interference be justified on christian principles? "In sorrow thou shalt bring forth," was the decree of the Creator, and how, it is urged, can you render labor painless without a presumptuous contravention of the Divine will? My answer is, that the principle, if followed out, would lead to a passive submission to all the physical ills which impend our state. The vaccine virus was just such a contravention, and precisely this objection was urged against it, as it had been previously urged against inoculation for small-pox. But I do not apprehend that physicians will interpose any such objections to the use of ether in obstetrics, and therefore dismiss this topic for others of greater practical moment.

Baron Dubois, as has been seen, does not believe that the agent will become universally, or even generally applicable to ordinary midwifery practice. He is convinced that ether will be applicable to but a limited number of peculiar cases.

Professor Simpson, of Edinburgh, is much more sanguine in his views. He has pushed his experiments with great industry, and some months ago, it is stated, had used the agent in fifty cases, and in every one with perfect safety and success. Certainly, therefore, so far, the facts are in favor of his opinion. Not only in his cases, but in all the cases published, the relief from pain has been great and almost instantaneous; and, at the same time, no untoward accident has attended the ether's use. It has been alleged, that hemorrhage would be more likely to ensue; but as the uterus is found to contract as well when it is used as when it is not, I do not see why this should be so. Convulsions were apprehended; but in all the recorded cases no such accidents followed its administration.

Dubois bases his opinion that ether can never come into general use upon two principal grounds; 1st, the small number of cases which had fallen under his observation; and 2d, the impossibility, or probable danger of keeping a woman etherized for several hours. But Professor Simpson kept up the etherization in some of his cases for hours, and other obstetricians have pursued the same practice. Dr. Protheroe Smith, of London, reports cases as favorable to the practice as those of Prof. Simpson, and is quite as warm an advocate for the use of ether in ordinary cases of labor. He closes an exceedingly interesting lecture on the subject with the following remarks:

"In conclusion, I would state it as my opinion, that with pefectly pure ether, carefully administered by skilful persons, and with good apparatus, and especially by one containing an appendage with a supply of oxygen, the operation not being commenced until efficient etherization is produced, the employment of ether is not only justifiable, but promises to be instrumental in materially diminishing the dangers of operative midwifery. Probably, in natural cases it will be both sufficient and safer to carry the etherization only to the second stage, in which partial consciousness remains, but sensation is abolished; and towards the end, when the pains are ordinarily intolerable, to induce perfect narcotism. From the results which I have already obtained, it is my intention to continue the use of this valuable agent, and I do not hesitate to state my belief, that future experience will fully confirm my present opinion."

Dr. Smith, in the same lecture, refers to other applications of ether in forms of disease allied to obstetrics, and relates that in several cases of dysmenorrhœa it acted like magic. A case of puerperal mania, he informs us, was immediately and permanently relieved by it; and late Journals make mention of cases of neuralgia, colica pictonum, and asthma, in which it was used with striking success.

Is the administration of ether attended with no danger? This is a grave question, and gravely to be pondered. We have seen that Baron Dubois does not look upon the agent as devoid of powers to do mischief. In all his cases, he believes it was innoxious, and it appears to have been equally so in the hands of Professor Simpson and Dr. Smith. Still, who can say, that however harmless, hitherto, it will never prove disastrous in its action? Time only can determine such a question. Observations must be greatly multiplied before we can be firm in such an assurance. Meantime, by oxygen and electricity, practitioners are preparing themselves against the casualties likely to arise in cases of its administration.

Paris, July 30th, 1847.

